# A breed-of-origin of alleles model that includes crossbred data improves predictive ability for crossbred animals in a multi-breed population

**DOI:** 10.1186/s12711-023-00806-1

**Published:** 2023-05-15

**Authors:** Ana Guillenea, Mogens Sandø Lund, Ross Evans, Vinzent Boerner, Emre Karaman

**Affiliations:** 1 Center for Quantitative Genetics and Genomics, Aarhus University, 8000 Aarhus C, Denmark; 2 ICBF, Link Road, Carrigrohane, Ballincollig, Co. Cork, P31 D452 Ireland

## Abstract

**Background:**

Recently, crossbred animals have begun to be used as parents in the next generations of dairy and beef cattle systems, which has increased the interest in predicting the genetic merit of those animals. The primary objective of this study was to investigate three available methods for genomic prediction of crossbred animals. In the first two methods, SNP effects from within-breed evaluations are used by weighting them by the average breed proportions across the genome (BPM method) or by their breed-of-origin (BOM method). The third method differs from the BOM in that it estimates breed-specific SNP effects using purebred and crossbred data, considering the breed-of-origin of alleles (BOA method). For within-breed evaluations, and thus for BPM and BOM, 5948 Charolais, 6771 Limousin and 7552 Others (a combined population of other breeds) were used to estimate SNP effects separately within each breed. For the BOA, the purebreds' data were enhanced with data from ~ 4K, ~ 8K or ~ 18K crossbred animals. For each animal, its predictor of genetic merit (PGM) was estimated by considering the breed-specific SNP effects. Predictive ability and absence of bias were estimated for crossbreds and the Limousin and Charolais animals. Predictive ability was measured as the correlation between PGM and the adjusted phenotype, while the regression of the adjusted phenotype on PGM was estimated as a measure of bias.

**Results:**

With BPM and BOM, the predictive abilities for crossbreds were 0.468 and 0.472, respectively, and with the BOA method, they ranged from 0.490 to 0.510. The performance of the BOA method improved as the number of crossbred animals in the reference increased and with the use of the correlated approach, in which the correlation of SNP effects across the genome of the different breeds was considered. The slopes of regression for PGM on adjusted phenotypes for crossbreds showed overdispersion of the genetic merits for all methods but this bias tended to be reduced by the use of the BOA method and by increasing the number of crossbred animals.

**Conclusions:**

For the estimation of the genetic merit of crossbred animals, the results from this study suggest that the BOA method that accommodates crossbred data can yield more accurate predictions than the methods that use SNP effects from separate within-breed evaluations.

**Supplementary Information:**

The online version contains supplementary material available at 10.1186/s12711-023-00806-1.

## Background

The use of crossbred animals in livestock production has been adopted for many years in pigs and poultry, and currently, it is increasing in cattle to improve the profitability of the systems [1]. Accordingly, there is an increasing interest in predicting the genetic merit of crossbred animals [2].

Genomic prediction (GP) consists of the use of a large number of single nucleotide polymorphisms (SNPs) to predict the genetic merit of selection candidates [3] and has become common in livestock due to the proven increase in the rate of genetic gain [4]. The accuracy of the estimation of SNP effects, and therefore of the prediction of genetic merit, relies heavily on the degree of linkage disequilibrium (LD) between the SNPs and the quantitative trait loci (QTL), which differs between breeds and may not be consistent across breeds [5]. This may challenge the estimation of the genetic merit of animals when reference populations involve multiple breeds. For example, the Irish national cattle genetic evaluation is based on a multi-breed population that includes many breeds (~ 40), and the vast majority of the animals are crossbreds with varying contributions from those pure breeds.

For prediction in crossbred animals, VanRaden et al. [6] suggested an approach based on partitioning the genetic merits of the crossbreds into breed-specific purebred terms, which rely on the estimates of SNP effects from separate within-breed genomic evaluations and breed proportions averaged across the genome, which are estimated from genotypes for each crossbred animal (referred by the authors as "Base Breed Representation", BBR). These average genomic breed proportions, which can take any value from 0 to 1 for a given breed in any individual, are then used as weights for combining the results obtained from separate within-breed genomic evaluations. Hence, all SNPs across the genome get equal weights when predicting an individual's genetic merit. Using simulations, Eiríksson et al. [7] reported that a method combining SNP effects that are estimated from separate within-breed genomic evaluations based on breed-of-origin of genome regions outperformed the method that was proposed by VanRaden et al. [6] across the four generations evaluated, with the largest difference observed in the first generation. The same methods were compared in a real dairy crossbred population, with similar results [8]. The method based on the breed-of-origin of genome regions used in Eiríksson et al. [7] relies on the estimation of the breed-of-origin (proportion) for each SNP allele (BOA), which, for a certain allele, can take only the values 0 or 1 when the allele can be assigned to a specific breed. Otherwise, the probability of BOA assignment can be considered. Following Eiríksson et al. [7], we will refer to the method of VanRaden et al. [6] as BPM and to that of Eiríksson et al. [7] as BOM. The limitation of both these methods is that the genetic merit of crossbred animals is predicted based only on purebred data. Karaman et al. [9] proposed a methodology that allows to include multiple breeds and crossbred animals in the reference population while accounting for BOA, and we will refer to this method as the breed-of-origin of alleles (BOA) method. Although both the BOM and BOA methods use the information on breed-of-origin of alleles for crossbred selection candidates, BOM does not consider crossbred animals in the reference population, whereas the BOA does. On the one hand, the inclusion of crossbred animals in the reference population may increase the accuracy of GP by the addition of intra-breed information. On the other hand, a joint analysis of all the breeds' data may help to transfer information between breeds if SNP effects are allowed to be correlated in the BOA method.

Thus, this study had two principal aims: (1) to investigate whether the accuracy of GP from a BOA method is higher than that from methods that combine SNP effects from within-breed evaluations (BPM and BOM) for crossbred animals and (2) to investigate the impact of the number of crossbred animals in the reference population when using the BOA method. The predictive ability and absence of bias of the three methods were tested using purebred and crossbred animals from the multi-breed Irish cattle population.

## Methods

### Data

The cattle data used in our study were sourced from the Irish Cattle Breeding Federation (ICBF, https://www.icbf.com) national database. In the genomic prediction models, we used carcass weight (kg) and genomic data from 26,164 purebred and 47,818 crossbred animals born between 2000 and 2020 and slaughtered between 307 and 1277 days of age. In this study, an animal was defined as a purebred when the pedigree breed proportion was 32 out of 32 from a particular breed, which means that all its ancestors, five generations back, had to be registered as a purebred of the same breed; otherwise, it was considered a crossbred.

Marker data were collected and pre-processed by the ICBF. Crossbred animals were genotyped with 12 SNP arrays, of which 98% were of medium density (between 50 and 54K), while purebred animals were genotyped with 20 SNP arrays, of which 84% were of medium density (between 45 and 63K). The ICBF provided the genotypes imputed to a common reference panel for all breeds and phased them into two haplotypes using the FImpute software [10]. In total, 50,493 SNPs across the 29 bovine autosomes were available for statistical analysis.

### Estimation of breed-of-origin

Analysis using BOA information requires that the breed-of-origin of phased alleles in the crossbred animals is known. We estimated BOA for all the crossbred animals using the four breeds that contributed the most to the crossbred genotyped animals as parental breeds, i.e. Limousin, Charolais, Angus and Holstein, and then Others group that included the purebred animals of the five breeds that ranked after the previous four in terms of their contribution to the crossbreds, i.e. Friesian, Simmental, Hereford, Belgian Blue and Shorthorn. We used this grouping strategy because the number of purebred animals from these breeds was small, and their individual contribution to the crossbred animals was very low (~ 3%). The contribution of the other breeds to the crossbreds was so low that they were considered negligible.

We used the AllOr software [7] to infer the breed-of-origin of each SNP allele of all pre-selected genotyped crossbred animals (166,925). This software was designed to detect BOA in genotypes of crossbred animals where the dam is a purebred or crossbred from known breeds, and the sire is a purebred from a known breed, and it was later extended to allow the use of crossbred sires (Jon Eiríksson, personal communication). AllOr requires (i) the phased genotypes of the representative samples of all contributing pure breeds, (ii) the phased genotypes of the crossbred animals, (iii) a pedigree file connecting the crossbred animals to genotyped purebred ancestors (optional), and (iv) information on sire and dam breeds of the crossbred animals. Details of the procedures for the assignment of alleles in AllOr are described in Eiríksson et al. [7]. The genotypes from the pure breeds used in AllOr consisted of 32,801 animals, of which 24,000 were Limousin, Charolais, Angus and Holstein (i.e. 6000 for each breed), 8000 were Simmental, Hereford, Belgian Blue and Shorthorn (i.e. 2000 for each breed) and 801 were Friesian. To assign BOA, the entire set of crossbreds was divided into 10 sets, of which nine contained 16,000 animals (16K), and one contained 22,925 animals.

We also included a pedigree file that contained 481,058 animals born from 1966 to 2020 (40,869 purebreds), which were obtained by tracing back the genotyped crossbred animals in a full pedigree containing 11,850,818 animals. We used the DMU Trace program [11], with a default pruning value (i.e. GPRUNE = 0), in which the pedigree is not pruned to a specific number of generations, but the pruning is done based on non-informative individuals.

AllOr detects BOA for each chromosome separately, thus requiring a split analysis by chromosome. Therefore, 290 analyses were performed (one per chromosome per set). On average, across the 10 sets, for the longest chromosome (3139 SNPs), it took ~ 102 h to detect BOA, and for the shortest chromosome (895 SNPs), it took ~ 28 h. Analyses required ~ 860 Mb and ~ 283 Mb of memory for the longest and the shortest chromosomes, respectively.

The alleles with their assigned BOA were used to build the breed-specific genotype matrices required for the BOA method and for validation using the BOM (explained later). The alleles not assigned to Limousin, Charolais, Angus and Holstein, and Others were also defined as originating from the Others group.

The contribution of Angus and Holstein alleles in the crossbred genotyped population was small, i.e. ~ 13% for each breed and only 2215 Angus and 1515 Holstein individuals had phenotype data in addition to genotypes. Therefore, keeping these as two separate breeds when estimating parameters in the models resulted in convergence issues, especially in the estimation of covariances between breeds in the BOA method. Thus, these breeds were included in the Others group, keeping three components for GP, the two main breeds: Limousin and Charolais, and Others.

After estimating BOA, genomic breed proportions (GBP) were calculated for each crossbred animal by counting the alleles assigned to Limousin, Charolais, or the Others group.

### Models

Before running the prediction models, the phenotypes of all animals in the population (10,159,498) were pre-corrected with the DMU software [12] using an animal model that resembles the routine genetic evaluation for carcass weight in Ireland as carried out by the ICBF. The model included the following fixed effects: birth year, type of birth (twin or single), slaughterhouse, and age at slaughter linear, quadratic and cubic; and the following random effects: contemporary group of herd of slaughter and prior to slaughter, dam permanent environment, and animal genetic effect. The adjusted phenotypes were then calculated as the summation of the residuals of the model and the animal genetic effects. Unlike the current ICBF model, which includes effects to correct for heterosis and recombination losses, we did not include non-additive effects to maintain the effects that are partially captured by the additive effect when crossbred animals are used in the BOA method.

We compared three methods based on different data sources in the reference population and validation strategies used. In two methods (BPM and BOM), separate within-breed evaluations were performed to obtain SNP effects for each breed. Those effects were then used to obtain the PGM, either (1) based on GBP (BPM) or (2) based on breed-origin-of-alleles (BOM). The third method (BOA) considered information on crossbred animals to estimate SNP effects and combined those effects with the BOA estimates to obtain PGM. The BOA method was implemented assuming that the SNP effects of the different breeds are (3a) uncorrelated (BOA_UNCOR_) or (3b) correlated (BOA_COR_), as shown in Table 1. It should be noted that these correlations were not fixed but were estimated, as explained later.

**Table 1:** Characteristics of each method

Method	Analysis to estimate SNP effects	Reference	SNP effects
BPM	3 single breed	P	Not correlated
BOM	3 single breed	P	Not correlated
BOA_UNCOR_	1 multi-breed	P + C	Not correlated
BOA_COR_	1 multi-breed	P + C	Correlated

P: purebred; C: crossbred

### Estimation of SNP effects

#### BPM and BOM

For each breed, separately (Charolais, Limousin and Others), we applied a Bayesian whole-genome regression method to estimate SNP effects:

1 $${\mathbf{y}}^{*} = \boldsymbol{1}{\upmu } + {\mathbf{Zu}} + {\mathbf{e}},$$

where ${\mathbf{y}}^{*}$ is the vector of adjusted phenotypes for the reference animals, $\boldsymbol{1}$ is a vector of 1s, $\upmu$ is the overall mean, $\mathbf{Z}$ is the matrix of centered genotypes based on current allele frequencies in the reference population, $\mathbf{u}$ is the vector of SNP effects, and $\mathbf{e}$ is the vector of random residuals. The vector of SNP effects was assigned a prior of a normal distribution $\mathbf{u}|{\upsigma }_{\mathrm{u}}^{2}\sim \mathrm{N}\left(\boldsymbol{0},\mathbf{I}{\upsigma }_{\mathrm{u}}^{2}\right)$, where $\mathbf{I}$ is an identity matrix and ${\upsigma }_{\mathrm{u}}^{2}$ is the variance of SNP effects. The ${\upsigma }_{\mathrm{u}}^{2}$ was further assigned a scaled inverted chi-square prior, with a degree of freedom (${df}_{u}$) and a scale parameter (${S}_{u}$), where ${df}_{u}=4$ and ${S}_{u}=\frac{{\upsigma }_{{\mathrm{u}}_{old}}^{2}\left({df}_{u}-2\right)}{{df}_{u}}$ [13]. Here, ${\upsigma }_{{\mathrm{u}}_{old}}^{2}$ is the variance of SNP effects that was estimated in an earlier analysis. This Bayesian regression method given above is equivalent to SNP best linear unbiased prediction (SNPBLUP), except that marker and residual variances are treated as unknown and estimated, whereas they are assumed to be known in SNPBLUP. For residual variances, ${df}_{e}=4$ and ${S}_{e}=\frac{{\sigma }_{{e}_{old}}^{2}\left({df}_{e}-2\right)}{{df}_{e}}$, where ${\upsigma }_{{e}_{old}}^{2}$ is the residual variance that was also estimated in an earlier analysis.

#### BOA_COR_ and BOA_UNCOR_

The following model was used to jointly analyze all purebreds and crossbred animals, which is referred to as the BOA model [9]:

2 $${\mathbf{y}}^{*} = \boldsymbol{1}{\upmu } + {\mathbf{Xb}} + {\mathbf{Z}}_{{{\text{CH}}}} {\mathbf{u}}_{{{\text{CH}}}} + {\mathbf{Z}}_{{{\text{LM}}}} {\mathbf{u}}_{{{\text{LM}}}} + {\mathbf{Z}}_{{{\text{OT}}}} {\mathbf{u}}_{{{\text{OT}}}} + {\mathbf{e}},$$

where ${\mathbf{y}}^{\mathbf{*}}$, $\boldsymbol{1}$, $\upmu$, $\mathbf{e}$ are as described for Eq. (1), $\mathbf{X}$ is the matrix of GBP calculated after the BOA estimation step, and $\mathbf{b}$ is the vector of fixed breed effects, ${\mathbf{Z}}_{\mathrm{CH}}$, ${\mathbf{Z}}_{\mathrm{LM}}$ and ${\mathbf{Z}}_{\mathrm{OT}}$ are the matrices of breed-specific content of SNPs for Charolais, Limousin, and Others, respectively. These $\mathbf{Z}$ (BOA matrices), were created as in Sevillano et al. [14]. In their general form, each BOA entry is ${x}_{jk}-{m}_{jk}{p}_{jk}$, where ${x}_{jk}$ is the partial genotype at locus $j$ originating from breed $k$, ${m}_{jk}$ is the number of alleles at locus $j$ originating from breed $k$, ${p}_{jk}$ is the breed-specific allele frequency for locus $j$ in breed $k$. The ${p}_{jk}$ was calculated as the number of occurrences of reference alleles generated from breed $k$ divided by the total number of alleles from breed $k$. An entry in the corresponding $\mathbf{Z}$ matrix is 0 when an animal carries no alleles at locus $j$ from a breed, 0-${p}_{jk}$ or 1-${p}_{jk}$ when an animal carries only one allele at locus $j$, and 0-2${p}_{jk}$, 1-2${p}_{jk}$ or 2-2${p}_{jk}$ when an animal carries both alleles at locus $j$ from breed $k$.

Finally, ${\mathbf{u}}_{\mathrm{CH}}$, ${\mathbf{u}}_{\mathrm{LM}}$ and ${\mathbf{u}}_{\mathrm{OT}}$ are the vectors of SNP effects for Charolais, Limousin, and Others, respectively. Each vector of SNP effects was assigned a prior in the form of a normal distribution: ${\mathbf{u}}_{\mathrm{k}}|{\upsigma }_{\mathrm{u},\mathrm{k}}^{2}\sim \mathrm{N}\left(\boldsymbol{0},\mathbf{I}{\upsigma }_{\mathrm{u},\mathrm{k}}^{2}\right)$, with $\mathrm{k}$ representing Charolais, Limousin, and Others. Consequently, the breed-specific SNP effects were assumed to be uncorrelated across breeds (BOA_UNCOR_).

The ${\upsigma }_{\mathrm{u},\mathrm{k}}^{2}$ were further assigned a scaled inverted chi-square prior, with a degree of freedom ($df$) and a scale parameter ($S$), where $df=4$ and ${S}_{k}=\frac{{\upsigma }_{{\mathrm{u}}_{old},\mathrm{k}}^{2}\left(df-2\right)}{df}$ [13]. Here, ${\upsigma }_{{\mathrm{u}}_{old},\mathrm{k}}^{2}$ is the SNP variance for breed $k$, estimated in an earlier analysis.

Priors were also assigned to estimate breed-specific SNP effects, but assuming that the SNP effects between the different breeds were correlated (BOA_COR_). In that case, a multivariate normal distribution was assigned for each vector of SNP effects: ${{\left[ \mathbf{u}_{\text{CH}}^{\prime}{{\mathbf{u}}_{\text{LM}}^{\prime}} \mathbf{u}_{\text{OT}}^{\prime} \right]}^{\prime}}| \mathbf{B}\sim N\left( 0,\mathbf{B}\otimes \mathbf{I} \right)$, where ${\mathbf{I}}$ is an identity matrix of size equal to the number of SNPs (50,493), and ${\mathbf{B}}$ is as follows [9, 15]:

$${\mathbf{B}} = \left[ {\begin{array}{*{20}c} {\sigma_{CH}^{2} } & {\sigma_{CH,LM} } & {\sigma_{CH,OT} } \\ {\sigma_{LM,CH} } & {\sigma_{LM}^{2} } & {\sigma_{LM,OT} } \\ {\sigma_{OT,CH} } & {\sigma_{OT,LM} } & {\sigma_{OT}^{2} } \\ \end{array} } \right].$$

The diagonals of ${\mathbf{B}}$ are the breed-specific SNP variances, while the off-diagonals are SNP covariances between the breeds. The ${\mathbf{B}}$ matrices were assumed to follow an inverted Wishart distribution with a shape $v_{B}$ and a scale ${\mathbf{V}}_{B}$ parameter: ${\mathbf{B}} | v_{B} , {\mathbf{V}}_{B} \sim IW\left( {v_{B} , {\mathbf{V}}_{B} } \right)$. ${\mathbf{V}}_{B}=({v}_{B}-3-1){\mathbf{B}}_{{\varvec{o}}{\varvec{l}}{\varvec{d}}}$, where ${v}_{B}=$ 6, and ${\mathbf{B}}_{{\varvec{o}}{\varvec{l}}{\varvec{d}}}$ is the matrix of SNP (co)variances obtained in an earlier analysis. The values of ${\upsigma }_{{\mathrm{u}}_{old}}^{2}$, ${\upsigma }_{{\mathrm{u}}_{old},\mathrm{i}}^{2}$ and ${\sigma }_{{e}_{old}}^{2}$ were obtained from a preliminary BOA analysis using animals in contemporary groups of herd of slaughter with at least 20 animals (5310 animals).

For all the models, the Markov-chain Monte Carlo (McMC) algorithm with Gibbs sampling was run for 100,000 cycles to infer model parameters, and the first 20,000 cycles were taken as the burn-in period and thus discarded. Every 10th cycle of the remaining samples was saved, giving 8000 posterior samples for each parameter. The mean value of each parameter over the posterior samples was used as its estimate. In addition to the estimates of SNP effects, we also obtained the posterior distribution of $\mathbf{B}$, which was used to estimate the correlation of SNP effects between breeds (Table 5). The analyses were conducted using in-house software tools written in the Julia programming language [16].

### Validation of methods

Reference and validation populations were defined similarly to those in Su et al. [17] and Liu et al. [18] to reduce the relationship between reference and validation populations. First, we set an initial threshold of 2018 to define the validation population as the animals born after that year. Second, if more than half of the offspring was born after 2018 for a bull, the entire group (bull and offspring) was assigned to the validation population; otherwise, it was assigned to the reference population. Hence, each bull was included with its offspring in the same population.

For the reference set used in within-breed evaluations, we used all available purebred animals that were not allocated to the validation population: 5948 Charolais, 6771 Limousin and 7552 Others. For the reference sets used in the BOA method, we used the purebreds mentioned for within-breed evaluations and added an increasing number of crossbred animals. The selection of the crossbred animals to be included was based on a minimum number of animals in each contemporary group of the herd of slaughter: 15 animals per group (4423), 10 animals per group (8239) and five animals per group (18,385). A summary of the reference sets used in the BOA model is in Table 2.

**Table 2:** Summary of animals used as reference set in the different BOA scenarios

Scenario	Purebreds (P)	Crossbreds (C)	Total
20K_P + 4K_C	20,271	4423	24,694
20K_P + 8K_C	20,271	8239	28,510
20K_P + 18K_C	20,271	18,385	38,656

The validation set consisted of 35,362 animals, of which 5893 were purebred (1117 Charolais, 1670 Limousin, and 3106 Others) and 29,433 were crossbred. Crossbred animals were separated into four groups depending on the contribution of the two main breeds: a contribution of < 25% (6343 animals), $\ge$ 25 to < 50% (3360 animals), $\ge$ 50 to < 85% (10,670 animals) and $\ge$ 85% (9060 animals).

After estimating the SNP effects using each reference population, PGM were estimated differently depending on the approach used. It should be noted that both BPM and BOM rely on the same set of SNP effect estimates obtained by fitting Eq. (1) to each breed’s data alone. Thus, a comparison of the PGM for purebred animals is irrelevant between BPM and BOM.

#### BPM

The PGM from the BPM is obtained as follows [6]:

3 $${\text{PGM}}_{{{\text{BPM}},i}} = {\text{GBP}}_{{{\mathbf{CH}},i}} {\mathbf{m}}_{i}^{\prime} {\hat{\mathbf{u}}}_{{\boldsymbol{1}_{{{\text{CH}}}} }} + {\text{GBP}}_{{{\mathbf{LM}},i}} {\mathbf{m}}_{i}^{\prime} {\hat{\mathbf{u}}}_{{\boldsymbol{1}_{{{\text{LM}}}} }} + {\text{GBP}}_{{{\mathbf{OT}},i}} {\mathbf{m}}_{i}^{\prime} {\hat{\mathbf{u}}}_{{\boldsymbol{1}_{{{\text{OT}}}} }} + \sum\limits_{{k = 1}}^{{N_{k} }} {{\text{GBP}}_{{k,i}} {{\hat{\text{b}}}}_{{\text{k}}} },$$

where ${\mathrm{GBP}}_{\mathrm{CH},i}$, ${\mathrm{GBP}}_{\mathrm{LM},i}$ and ${\mathrm{GBP}}_{\mathrm{OT},i}$ are the GBP values for Charolais, Limousin and Others, respectively, on animal $i$, ${\mathbf{m}}_{i}$ is the vector of allele contents for animal $i$, ${\hat{\mathbf{u}}}_{{{\boldsymbol{1}}_{{{\text{CH}}}} }}$, ${\hat{\mathbf{u}}}_{{{\boldsymbol{1}}_{{{\text{LM}}}} }}$ and ${\hat{\mathbf{u}}}_{{{\boldsymbol{1}}_{{{\text{OT}}}} }}$ are the vectors of SNP solutions from Eq. (1) for Charolais, Limousin and Others, respectively, ${\text{GBP}}_{k,i}$ is the GBP value for breed $k$ on animal $i$, and ${\widehat{\mathrm{b}}}_{\mathrm{k}}$ is the mean effect of breed $k.$

#### BOM

The PGM from the BOM is obtained as follows [7]:

4 $${\text{PGM}}_{{{\text{BOM}},i}} = {\mathbf{z}}_{{\text{CH},i}}^{^{\prime}} {\hat{\mathbf{u}}}_{{\boldsymbol{1}_{{{\text{CH}}}} }} + {\mathbf{z}}_{{{\text{LM}},i}}^{^{\prime}} {\hat{\mathbf{u}}}_{{\boldsymbol{1}_{{{\text{LM}}}} }}+ {\mathbf{z}}_{{{\text{OT}}i}}^{^{\prime}} {\hat{\mathbf{u}}}_{{\boldsymbol{1}_{{{\text{OT}}}} }} + \sum\limits_{k = 1}^{{N_{k} }} {{\text{GBP}}_{k,i} {\hat{\text{b}}}_{{\text{k}}} ,}$$

where ${\mathrm{GBP}}_{k,i}$, ${\widehat{\mathrm{b}}}_{\mathrm{k}}$ and ${\hat{\mathbf{u}}}_{{{\boldsymbol{1}}_{{{\text{CH}}}} }}$, ${\hat{\mathbf{u}}}_{{{\boldsymbol{1}}_{{{\text{LM}}}} }}$ and ${\hat{\mathbf{u}}}_{{{\boldsymbol{1}}_{{{\text{OT}}}} }}$ are as defined for Eq. (3), ${\mathbf{z}}_{{{\text{CH}},i}}$, ${\mathbf{z}}_{{{\text{LM}},i}}$ and ${\mathbf{z}}_{{{\text{OT}},i}}$ are the vectors of breed-specific allele contents for Charolais, Limousin and Others, respectively for animal $i$.

#### BOA_UNCOR_ and BOA_COR_

The PGM from the BOA is obtained as follows:

5 $${\text{PGM}}_{{{\text{BOA,}}i}} = {\mathbf{z}}_{{{\text{CH,}}i}}^{^{\prime}} {\hat{\mathbf{u}}}_{{\boldsymbol{2}_{{{\text{CH}}}} }} + {\mathbf{z}}_{{{\text{LM,}}i}}^{^{\prime}} {\hat{\mathbf{u}}}_{{\boldsymbol{2}_{{{\text{LM}}}} }} + {\mathbf{z}}_{{{\text{OT,}}i}}^{^{\prime}} {\hat{\mathbf{u}}}_{{\boldsymbol{2}_{{{\text{OT}}}} }} + \sum\limits_{k = 1}^{{N_{k} }} {{\text{GBP}}_{k,i} {\hat{\text{b}}}_{{\text{k}}} },$$

where ${\mathrm{GBP}}_{k,i}$ and ${\widehat{\mathrm{b}}}_{\mathrm{k}}$ are as defined for Eq. (3), ${\mathbf{z}}_{{{\text{CH}},i}}$, ${\mathbf{z}}_{{{\text{LM}},i}}$ and ${\mathbf{z}}_{{{\text{OT}},i}}$ are as described for Eq. (4) and ${\hat{\mathbf{u}}}_{{\boldsymbol{2}_{{{\text{CH}}}} }}$, ${\hat{\mathbf{u}}}_{{\boldsymbol{2}_{{{\text{LM}}}} }}$ and ${\hat{\mathbf{u}}}_{{\boldsymbol{2}_{{{\text{OT}}}} }}$ are the vectors of SNP solutions from Eq. (2) for Charolais, Limousin and Others, respectively.

### Predictive ability

We tested the ability of each method to predict the performance of the validation animals. The predictive abilities of each method were calculated as the correlation between adjusted phenotypes (y*) and PGM for the validation animals. A nonparametric bootstrap procedure, with a size equal to that of the validation population, was obtained by random sampling with replacement from validation animals. We repeated the bootstrap procedure to obtain 10,000 bootstrap samples of the PGM of each scenario. The comparisons were performed using a two-tailed paired t-test of the 10,000 samples of each scenario. Bonferroni correction was applied to account for the multiple-test comparisons. The slopes of regression of adjusted phenotypes on PGM were calculated as a measure of bias for all methods. Slopes of ~ 1 imply no bias, while slopes greater than or less than 1 indicate under- or overdispersion of genetic merits, respectively.

## Results

### Breed-of-origin assignment

**Fig. 1:**
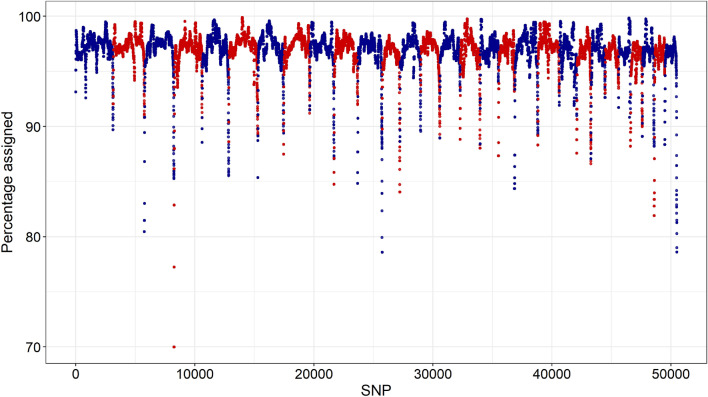
Average proportion of alleles assigned to breeds across the genome. Alternate red and blue refer to each bovine autosome

The AllOr software was able to assign most alleles to their breed-of-origin, i.e. on average, 97.03% (ranging from 69.99 to 99.88%) of the alleles were assigned a breed-of-origin. We observed no trend in the assignment between different chromosomes, but the extremities of all the chromosomes had the lowest assignment (Fig. 1).

### Genomic prediction

The genomic prediction results are in Table 3. The BOA method enhanced predictive ability over both BPM and BOM for crossbred animals, while for the last two there was no difference in predictive ability. The BOA method, on average, outperformed the BPM by 5%, 7%, and 8% when ~ 4K, ~ 8K and ~ 18K crossbred animals were included in the reference population. There were no significant differences between methods for purebred animals. The results of BOA_COR_ and BOA_UNCOR_ did not differ significantly. However, within the BOA analyses, BOA_UNCOR_ including ~ 4K crossbreds yielded the worst performance and the BOA_COR_ including ~ 18K crossbreds yielded the best performance.

**Table 3:** Predictive ability (standard error) for each method with a significant test

	BPM	BOM	BOA					
			UNCOR	COR	UNCOR	COR	UNCOR	COR
	20K_P		20K_P + 4K_C		20K_P + 8K_C		20K_P + 18K_C	
**Crossbred**								
All	0.468^c^ (0.005)	0.472^c^ (0.005)	0.490^b^ (0.005)	0.497^ab^ (0.005)	0.495^ab^ (0.005)	0.502^ab^ (0.005)	0.502^ab^ (0.004)	0.510^a^ (0.004)
By bp								
< 25%	0.358^c^ (0.011)	0.349^c^ (0.011)	0.393^abc^ (0.011)	0.401^ab^ (0.011)	0.397^abc^ (0.011)	0.406^ab^ (0.011)	0.396^abc^ (0.011)	0.409^a^ (0.011)
$\ge$25–< 50%	0.261^bc^ (0.017)	0.253^c^ (0.016)	0.326^ab^ (0.015)	0.343^a^ (0.015)	0.340^a^ (0.015)	0.355^a^ (0.015)	0.357^a^ (0.015)	0.373^a^ (0.015)
$\ge$50–< 85%	0.246^e^ (0.009)	0.264^de^ (0.009)	0.290^bcd^ (0.009)	0.301^abc^ (0.009)	0.297^abc^ (0.009)	0.310^ab^ (0.009)	0.318^ab^ (0.009)	0.331^a^ (0.009)
$\ge$85%	0.238^d^ (0.010)	0.258^ cd^ (0.010)	0.279^bcd^ (0.010)	0.293^abc^ (0.010)	0.292^abc^ (0.010)	0.304^ab^ (0.010)	0.317^ab^ (0.010)	0.327^a^ (0.010)
**Purebred**								
Charolais	0.225^a^ (0.029)		0.245^a^ (0.029)	0.252^a^ (0.029)	0.253^a^ (0.029)	0.259^a^ (0.029)	0.273^a^ (0.029)	0.279^a^ (0.029)
Limousin	0.285^a^ (0.023)		0.306^a^ (0.023)	0.300^a^ (0.024)	0.313^a^ (0.023)	0.309^a^ (0.023)	0.316^a^ (0.023)	0.314^a^ (0.023)

Values with different superscripts indicate significant differences at P < 0.05 with Bonferroni correction, within each row; predictive abilities of BPM, BOM, BOA_UNCOR_ and BOA_COR_ were compared to each other within the same group of animals

bp: breed proportion of the two main breeds

The advantage of BOA over the other two methods for crossbred animals tended to be larger as the proportion of the main breeds contained in the tested crossbred animals increased. The increases with the BOA_COR_ in the 20K_P_18K_C scenario compared to those of BPM were equal to 14%, 43%, 35% and 37% for the groups with a contribution of the main breeds of < 25%, $\ge$ 25 to < 50%, $\ge$ 50 to < 85% and $\ge$ 85%, respectively. There was no significant difference between BPM and BOM, regardless of the contribution from the main breeds. The predictive abilities for the Limousin were slightly higher than those for the Charolais although the significance between these differences was not tested. For purebred performance, BPM and BOM share the same set of SNP effect estimates, and therefore, the difference between them lies only in the prediction of the crossbred animals' genetic merit. For purebred performance, there was no significant difference between these methods and BOA.

### Prediction bias

The regression slopes of adjusted phenotypes on PGM per scenario and method are in Table 4. The regressions can be seen as a measure of the bias of PGM. Slopes smaller than 1 indicate overdispersion of PGM, whereas slopes larger than 1 indicate an underprediction of PGM. All methods showed overdispersion of PGM for crossbred animals, but the slopes of regression for the BOA method were closer to 1 than for the other two methods. The increase in the number of crossbred animals tended to yield slopes approaching 1. For the groups divided by breed proportions of the two main breeds, BPM showed an underprediction of the PGM for animals with a breed composition of the main breeds lower than 50%, while the results were the opposite for the groups of animals with more than 50% of the main breeds. For methods considering the origin of the genomic segments for crossbred selection candidates (BOM and BOA), the slopes tended to be smaller (more overdispersion) as the contribution of the main breeds increased. For the Charolais animals, slopes indicated an overdispersion of the PGM across all methods and scenarios but tended to be closer to 1 as the number of animals increased in the analysis using BOA. On the contrary, for the Limousin animals, slopes showed underdispersion of the PGM across all methods and scenarios, but the increase in the number of animals also tended to show slopes closer to 1.

**Table 4:** Average slope of regression for predicted genetic merits on adjusted phenotypes (standard error) for each method

	BPM	BOM	BOA					
			UNCOR	COR	UNCOR	COR	UNCOR	COR
	20K_P		20K_P + 4K_C		20K_P + 8K_C		20K_P + 18K_C	
**Crossbred**								
All	0.752 (0.008)	0.704 (0.008)	0.784 (0.008)	0.785 (0.008)	0.814 (0.008)	0.790 (0.008)	0.926 (0.009)	0.853 (0.008)
By bp								
< 25%	1.102 (0.037)	1.067 (0.037)	1.073 (0.033)	1.071 (0.032)	1.044 (0.032)	1.034 (0.030)	1.025 (0.031)	1.017 (0.030)
≥25–< 50%	1.157 (0.075)	0.789 (0.052)	0.966 (0.048)	0.971 (0.046)	0.965 (0.047)	0.960 (0.044)	0.955 (0.042)	0.946 (0.041)
≥50–< 85%	0.871 (0.032)	0.692 (0.024)	0.725 (0.023)	0.719 (0.022)	0.727 (0.021)	0.758 (0.022)	0.758 (0.022)	0.738 (0.020)
$\ge$85%	0.908 (0.039)	0.763 (0.030)	0.743 (0.027)	0.727 (0.025)	0.744 (0.026)	0.729 (0.024)	0.759 (0.024)	0.730 (0.022)
**Purebred**								
Charolais	0.822 (0.109)		0.791 (0.094)	0.811 (0.093)	0.801 (0.093)	0.821 (0.092)	0.845 (0.093)	0.865 (0.091)
Limousin	1.008 (0.084)		1.050 (0.081)	1.039 (0.082)	1.047 (0.079)	1.033 (0.080)	1.034 (0.078)	1.025 (0.079)

bp: breed proportion of the main two breeds

### Correlations between SNP effects

The correlations between the SNP effects of the different breeds ranged from 0.49 to 0.86 (Table 5), computed from the posterior distribution of $\mathbf{B}$. The most stable correlation was between Charolais and Others, which on average was equal to 0.72 and remained almost constant as the number of crossbred animals in the dataset increased. The correlation between Charolais and Limousin ranged from 0.69 to 0.86 and tended to decrease as the number of crossbred individuals in the reference population increased. The correlations between Limousin and Others ranged from 0.49 to 0.65, with no clear trend. We observed that as the number of crossbred animals increased, the correlations involving the Others group were estimated with higher precision (lower standard error). These correlations between the SNP effects of the breeds reflect the genetic correlations between them [19].

**Table 5:** Estimated correlation (standard error) of SNP effects between groups calculated from BOA_COR_

	Charolais-Limousin	Charolais-Others	Limousin-Others
20K_P + 4K_C	0.86 (0.06)	0.71 (0.07)	0.49 (0.11)
20K_P + 8K_C	0.85 (0.06)	0.73 (0.07)	0.65 (0.08)
20K_P + 18K_C	0.69 (0.07)	0.73 (0.05)	0.56 (0.06)

## Discussion

In this study, we investigated the use of three methods for predicting the genotypic merit of crossbred animals. On the one hand, the first two methods consider only purebred information (phenotypes and genotypes) to estimate SNP effects and combine those SNP solutions based either on breed proportions (BPM) or on the breed-of-origin of the genome regions (BOM). On the other hand, the third method relies not only on the purebred but also on the crossbred information and takes the breed-of-origin of the genome regions into account in the analysis (BOA method). In addition, for the last method, the impact of including different numbers of crossbred animals in the reference population was investigated.

The BOM and BOA methods require that alleles are traced back to their breed-of-origin with high accuracy. The BOM method uses this information only to predict the genetic merit of crossbred animals but not to estimate the SNP effects, whereas the BOA method also uses it to integrate crossbred data in the reference population and thereby to estimate the SNP effects. Errors in the breed-of-origin assignments can affect the performance of both the BOM and BOA methods, but such errors may have a greater impact on the BOA method, as they also impact the accuracy in the estimation of the SNP effects. If the origins of alleles at certain loci are switched (e.g., from Charolais to Limousin or vice versa), then the estimates of breed-specific alleles will be affected by information from the opposite breed, resulting in a higher similarity, and therefore, in a higher correlation between them [20], when the BOA method is used. For crossbred animals with a more complex breed composition, the number of alleles wrongly assigned can be larger, and thus more overestimation of the correlations is likely to occur [20].

VanRaden et al. [6] proposed a simpler approach to predict the PGM of crossbred animals, which avoids tracing the alleles to their breed-of-origin. In the approach of VanRaden et al. [6], SNP effects from separate within-breed evaluations are combined using BBR to measure the proportion of genome originating from each breed in the crossbred animal. The BPM method here and the one used by Eiríksson et al. [7] are based on the approach presented in VanRaden et al. [6] but with some differences. In Eiríksson et al. [7], GBP were estimated from all marker genotypes with a linear Gaussian model rather than assuming a heavy-tailed distribution as in VanRaden et al. [20]. In addition to accounting for the genome proportions from each breed, Eiríksson et al. [7] calculate the BBR from genotypes with some differences from what was proposed by VanRaden et al. [6]. In the BPM method applied here, the proportions of the genome from each breed (GBP) were estimated based on BOA assignments and used for both BPM and BOM.

A high percentage of the alleles were assigned to a breed-of-origin (i.e. only 3% were unassigned) even when the type of crosses varied in the population. The assignments did not differ much between chromosomes, but their extremities presented lower assignments than the middle regions. This occurs with haplotype-based assignment methods because the number of possible matching haplotypes decreases when the algorithm looks only in one direction, which occurs at the start and end of the chromosomes [7]. For a rotational crossbred simulated population (using purebred sires), the same software assigned 99.8% of the alleles [7]. For a real population, including simple crosses of Holstein, Jersey, and Red Dairy Cattle (i.e., first-generation crosses, three-way crosses, and backcrosses primarily), it assigned 99.3% of the alleles [8]. In our study, some alleles that could not be assigned to a specific breed had a 75% chance of originating from Limousin or Charolais; others had a 50% chance of originating from one of these breeds. In this study, these alleles were assigned to the Others group. In the BOM method proposed by Eiríksson et al. [7], probabilities (values between 0 and 1) were assigned to alleles that could not be assigned to a certain breed instead of assigning them to the Others group as in the BOM applied here. Taking into account the probabilities in the assignments has already been reported to improve prediction accuracies using the BOA method [21].

The BOA method yielded a higher predictive ability for crossbred animals than the two BPM and BOM methods that are based on combining SNP solutions from separate within-breed evaluations, which indicates that SNP effects are estimated with higher accuracy when crossbred data are included in the reference population with the help of BOA. In addition, the predictive ability for crossbreds increased as the number of crossbred animals in the reference population increased. In our study, the predictive abilities of the methods that use SNP solutions from separate within-breed evaluations were not significantly different in most cases, while the BOM method proposed by Eiríksson et al. [7] outperformed BPM in simulations and real data [7, 8]. This may be due to the fact that in our study, GBP were calculated based on allele assignments, while Eiríksson et al. [7] calculated them based on a regression on the markers of the pure breeds. From these GBP, they calculated BBR, which was restricted to sum to 1 across breeds and to have a value between 0 and 1 for any breed, as described in VanRaden et al. [6]. In that sense, our BPM and BOM are more similar because for each animal the breed composition is strictly the same in both models, while they may differ in their models.

In addition to assessing the methods' ability to predict the genetic merits of crossbred animals, we also assessed their predictive ability in purebreds. Although there were no significant differences between the methods, BOA tended to yield higher predictive abilities for the purebred animals as the number of crossbreds in the reference population increased. When comparing the pure breeds, the predictive ability was higher for the Limousin than for the Charolais breed. One explanation is that the number of purebred animals to estimate SNP effects is larger for the Limousin than the Charolais breed (6771 vs 5948). Furthermore, when we used genomic relationship matrices within pure breeds, we observed that the genomic relationship between the reference and validation animals was higher in the Limousin than in the Charolais breed (results not shown), which could be due to the higher levels of artificial insemination used in this breed and thus implies that there were fewer effective sires in the Limousin population.

Using the BOA method, the number of SNP effects to be estimated increases as the number of breeds included in the analysis increases, whereas the number of phenotypes may be constant. In this study, for the proof of concept, we applied a restriction on the breed proportions of animals, taking two dominating breeds (Charolais and Limousin) in the population as the main breeds of interest and the rest of the breeds as Others, implicitly assuming that those in the Others group have the same SNP effects. Such decisions are also needed even for relatively simpler approaches [22].

The slopes of the BOA method were closer to 1 than those of BPM and BOM in most scenarios. The slope tended to be closer to 1 as the number of crossbred animals in the reference population increased. It has been previously reported that including crossbreds’ genomic information in prediction models reduces prediction bias [23]. The slopes of BPM and BOM were near 1 for the Limousin breed only, with the BOA method showing a slight underprediction for this breed. For Charolais, all methods showed overdispersion, with slopes for BOA slightly closer to 1.

When considering the methods based on breed-specific SNP effects, an important factor to consider is the level of genetic relationships among the breeds [24, 25], which can be measured from the correlation between SNP estimates. In this study, we reported moderate to relatively high correlations of the SNP effects of Charolais with those of Limousin. However, although these two beef breeds may cluster together in some phylogenetic analyses [26, 27], they have been extensively selected within breed, which may imply some genetic divergence. For example, a genome-wide association study involving Charolais and Limousin cows of the Irish national herd found only two SNPs located in both breeds within the same regions associated with carcass weight, cull-cow weight and live weight [28].

The amount of genetic variation explained may vary between regions leading to heterogeneous variance patterns across the genome [29]. Accounting for heterogeneous (co)variances along the genome has increased prediction reliability in Bayesian analysis for single-breed evaluations [30] and for admixed populations [20].

We ran additional BOA analyses (BOA_COR_ and BOA_UNCOR_) including only purebred animals in the reference population. Some covariances did not fully converge for the BOA_COR_, and thus we do not present those results. The predictive ability of the BOA_UNCOR_ using only purebred animals was 0.473 for crossbreds, 0.220 for Charolais and 0.287 for Limousin (results not given elsewhere), very similar to the results of BOM which also relies only on purebred data and assumes uncorrelated SNP effects.

In some way, the estimated allele substitution effects capture non-additive effects using the BOA method. First, breed differences that may be due to multi-locus interactions are accounted for by estimating separate SNP effects for each breed, which is similar to the separate within-breed evaluations. Second, the dominance is partially accounted for in the estimation of breed-specific allele substitution effects since they depend on the additive effect, the dominance effect and the allele frequencies [31]. More components could be added to the model to explicitly separate the additive from the non-additive effects.

The aim of the current study was to compare methodologies that are or can be implemented in multi-breed routine evaluations. From the comparisons performed here, it was not possible to differentiate if the increase in predictive ability of the BOA method was driven by accounting for the origin of the genome regions or by the use of additional information (i.e. crossbred data). To assess this, an additional joint analysis was performed using a combined dataset of the reference population animals, with just one additive component in the model (i.e. ignoring the BOA). The same three groups of animals were used as for the BOA method, 20K_P + 4K_C, 20K_P + 8K_C and 20K_P + 18K_C. The description of this model and the results are presented in Additional file 1 and Additional file 2: Tables S1 and S2. The results showed the same pattern as the BOA models, i.e., the predictive abilities increased as more crossbred animals were added to the reference population. However, the BOA_COR_ tended to yield higher predictive abilities than the joint analysis for crossbred animals, especially for animals with a contribution of 50% or more of the two main breeds. The BOA_COR_ led to the same predictive abilities as the joint analysis for Charolais but to slightly higher ones for Limousin (see Additional file 2: Table S1). There was no clear advantage of one method over the other in terms of bias of prediction (see Additional file 2: Table S2). A previous study using a subset of the Irish cattle population showed that when a pedigree BLUP model, a SNPBLUP model and the BOA method, all including the same purebred and crossbred data, were run, the inclusion of genomic data led to the greatest improvement for prediction of crossbreds, although the BOA method also outperformed the SNPBLUP [32]. The current multi-breed genetic evaluation carried out by the ICBF integrates genomic information, but the model ignores BOA. Our results showed that the predictions could be improved by using the BOA method.

For the current implementation of the BOA method, all crossbred animals need to be genotyped. The single-step genomic BLUP [33, 34] facilitates real-life situations where populations include genotyped and ungenotyped animals. This methodology has been modified to combine purebred and crossbred performances in two ways. One way is to use metafounders, which are pseudo-individuals included in the pedigree as founders without parent groups so that each metafounder represents one ancestral population [35]. Therefore, to include crossbred information, one metafounder is assigned per purebred line [36, 37]. The second way is based on breed-specific relationship matrices. It was proposed by Christensen et al. [38] by reformulating the model of Wei and van der Werf [39] and using breed-specific partial relationship matrices as in García-Cortés and Toro [40]. In this approach, partial relationship matrices describe relationships according to the genetic origin, and it adjusts the partial relationship matrices to be compatible with the pedigree-based relationship matrices. The BOA models can be extended to accommodate non-genotyped animals, either as a “SNP-effect” model where breed-specific SNP effects are estimated or equivalently as a "breeding value" model based on genomic relationship matrices, where genetic merits of individuals are predicted in one step [41]. The computational time and predictive performance of these alternative approaches need to be investigated. One of the limitations of the models based on BOA assignments is that, currently, allele assignment can be time-consuming. However, if genomic evaluations start to use the BOA method (or BOM) routinely, then inference of breed-of-origin of alleles is only needed for the newly genotyped crossbred animals. Nevertheless, efficient methods are needed for BOA assignment in large populations.

## Conclusions

Our results indicate that using phenotypes from crossbred and purebred animals can greatly enhance predictive ability over methods that rely on phenotypes from purebred animals only, even when ignoring BOA. Greater predictive abilities can be achieved by accounting for BOA. In addition, the predictive ability of the BOA method increases as the number of crossbred phenotypes included in the analysis increases. Therefore, this methodology could be a good alternative to predict genotypic values for crossbred selection candidates.

## Supplementary Information

**Additional file 1.** Model.

**Additional file 2: Table S1.** Predictive abilitywith standard errorbetween brackets and differences in % of the BOAUNCOR minus the joint analysis. Table S2. Title: Average slope of regression for LPGV on adjusted phenotypes with standardbetween brackets and differences in % of the BOACOR minus the joint analysis.

## Data Availability

The datasets supporting the conclusions of this article are property of ICBF. The software to run the BOA model is available in https://github.com/datasciencetoolkit/NextGP.jl.
