# Supplementary material for: A breed-of-origin of alleles model that includes crossbred data improves predictive ability for crossbred animals in a multi-breed population
Source: Genet Sel Evol. 2023 May 15;55:34. doi: 10.1186/s12711-023-00806-1 (PMC10184430; doi:10.1186/s12711-023-00806-1)
Supplement: Supplementary file 1 — Additional file 1. Model. [file 12711_2023_806_MOESM1_ESM.docx]

**Additional file 1**

**Model**

The joint model used to estimate SNP effects with both purebred and crossbred animal is as follows:

$\mathbf{y}^{\boldsymbol{*}}\mathbf{=1}\mu\mathbf{+Xb+Zu+e}$,

where $\mathbf{y}^{\boldsymbol{*}}$ is the vector of adjusted phenotypes for the reference animals (purebred and crossbred), $\mathbf{1}$ is the vector of 1s, $\mu$ is the overall mean, $\mathbf{X}$ is the matrix of GBP calculated from breed origin estimation, and $\mathbf{b}$ is the vector of fixed breed effects, $\mathbf{Z}$ is the matrix of centered genotypes based on current allele frequency in the reference animals, $\mathbf{u}$ is the vector of SNP effects, and $\mathbf{e}$ is the vector of random residuals. The vector of SNP effects was assigned a prior of a normal distribution $\mathbf{u}|\sigma_{u}^{2}\sim N\left( \mathbf{0,I}\sigma_{u}^{2} \right)$. The $\sigma_{u}^{2}$ was further assigned a scaled inverted chi-square prior, with a degree of freedom (df) and a scale parameter (S), where $df=4$ and $S_{i}=\frac{\sigma_{u_{old}}^{2}\left( df-2 \right)}{\mathrm{df}}$. Here, $\sigma_{u_{old}}^{2}$ is the SNP variance known from earlier analysis. For the residual variance ($\sigma_{e}^{2}$), ${df}_{e}=4$ and $S_{e}=\frac{\sigma_{e_{old}}^{2}\left( {df}_{e}-2 \right)}{{df}_{e}}$, where $\sigma_{e_{old}}^{2}$ is the residual variance also known from earlier analysis.
