# Supplementary material for: A breed-of-origin of alleles model that includes crossbred data improves predictive ability for crossbred animals in a multi-breed population
Source: Genet Sel Evol. 2023 May 15;55:34. doi: 10.1186/s12711-023-00806-1 (PMC10184430; doi:10.1186/s12711-023-00806-1)
Supplement: Supplementary file 2 — Additional file 2: Table S1. Predictive abilitywith standard errorbetween brackets and differences in % of the BOAUNCOR minus the joint analysis. Table S2. Title: Average slope of regression for LPGV on adjusted phenotypes with standardbetween brackets and differences in % of the BOACOR minus the joint analysis. [file 12711_2023_806_MOESM2_ESM.docx]

**Additional file 2 Table S1**

**Predictive ability (PA) followed by SE between brackets and differences in % of the BOA_COR_ model minus the joint model**

|  | **20K_P+4K_C** | | **20K_P+8K_C** | | **20K_P+18K_C** | |
| --- | --- | --- | --- | --- | --- | --- |
|  | **PA** | **diff (%)** | **PA** | **diff (%)** | **PA** | **diff (%)** |
| Crossbred | | | | | | |
| All | 0.490 (0.005) | 1 | 0.495 (0.005) | 1 | 0.509 (0.004) | 1 |
| By bp | | | | | | |
| < 25% | 0.393 (0.013) | 2 | 0.395 (0.013) | 2 | 0.406 (0.013) | 0 |
| 25-50% | 0.331 (0.017) | 5 | 0.346 (0.017) | 2 | 0.371 (0.016) | -1 |
| 50-85% | 0.290 (0.010) | 8 | 0.301 (0.010) | 7 | 0.334 (0.010) | 6 |
| > 85% | 0.285 (0.011) | 6 | 0.298 (0.011) | 6 | 0.329 (0.011) | 7 |
| Purebred | | | | | | |
| Charolais | 0.251 (0.029) | 0 | 0.259 (0.028) | 0 | 0.278 (0.028) | 0 |
| Limousin | 0.282 (0.024) | 3 | 0.290 (0.024) | 2 | 0.296 (0.024) | 3 |

bp: breed proportion of the two main breeds

**Additional file 2 Table S2**

**Average slope of regression for LPGVs on adjusted phenotypes followed by SE between brackets and differences in % of the BOA_COR_ model minus the joint model**

|  | **20K_P+4K_C** | **20K_P+8K_C** | **20K_P+18K_C** |
| --- | --- | --- | --- |
| Crossbred | | | |
| All | 1.002 (0.010) | 1.011 (0.010) | 1.034 (0.010) |
| By bp | | | |
| < 25% | 1.042 (0.035) | 1.017 (0.034) | 1.004 (0.032) |
| 25-50% | 0.958 (0.052) | 0.968 (0.049) | 0.987 (0.046) |
| 50-85% | 0.776 (0.027) | 0.789 (0.026) | 0.852 (0.025) |
| > 85% | 0.771 (0.032) | 0.778 (0.031) | 0.828 (0.029) |
| Purebred | | | |
| Charolais | 0.879 (0.101) | 0.883 (0.986) | 0.913 (0.095) |
| Limousin | 0.917 (0.080) | 0.909 (0.077) | 0.907 (0.075) |

bp: breed proportion of the two main breeds
